# Supplementary material for: CO oxidation activity of non-reducible oxide-supported mass-selected few-atom Pt single-clusters
Source: Nat Commun. 2020 Apr 20;11:1888. doi: 10.1038/s41467-020-15850-4 (PMC7171196; doi:10.1038/s41467-020-15850-4)
Supplement: Supplementary file 1 — Supplementary Information [file 41467_2020_15850_MOESM1_ESM.pdf]

Supplementary Information for

CO oxidation activity of non-reducible oxide-supported mass-selected few-atom Pt single-clusters

Beniya et al.

Corresponding Authors: Atsushi Beniya (beniya@mosk.tytlabs.co.jp)
Shougo Higashi (shigashi@mosk.tytlabs.co.jp)

Supplementary Notes

Supplementary Note 1.

Independency of the CO_2 formation temperature on the size of Pt_n/Al_2O_3.

Interestingly, the temperature of CO_2 formation is almost the same for the investigated clusters. Specifically, it is about 300 K, indicating that the same activation barrier exists for each Pt cluster, despite their very different structures **(Supplementary Note 2** and **Supplementary Fig. 4**). Similar results were obtained for Pt_n/MgO, over which CO_2 also formed at ~300 K, regardless of the number of atoms in each cluster [1]. Meanwhile, the same research group provided an interesting point of view, reporting that the cluster morphology changed during the CO oxidation reaction [2]. Such structural fluxionality, which was probably associated with the low binding energy of the clusters, could be the reason for this result.

Supplementary Note 2.

Activation energies of CO_2 formation and CO desorption for Pt_n/Al_2O_3.

This study revealed structural sensitivity for a single catalytic reaction event, but the steady-state reaction rate (r) depends not only on the amount of adsorbate but also on the activation energy (E_a), as shown in Supplementary Equation 1.

$$R = \nu \exp(-E_a/RT)\,\theta_{CO}\,\theta_O \qquad (1)$$

where ν, R, and T are the pre-exponential factor, gas constant, and temperature, respectively. Therefore, from the slopes of the corresponding Arrhenius plots[3,4], we tentatively estimated the activation energies for CO_2 formation and CO desorption as 6~8 and 40~60 kJ/mol, respectively (**Supplementary Fig. 4**). Furthermore, these values

were similar for the various Pt cluster sizes. Although our analyses might underestimate these values because the employed method does not consider the repulsive interactions between adsorbates[5,6], the value obtained for CO desorption is close to that for the steady-state reaction (60.3 kJ/mol)[7]. The estimated activation energy of CO desorption is constant regardless of the cluster size, suggesting that the reaction rate under steady-state conditions would also show a size dependency similar to that of the single catalytic reaction event.

Supplementary Note 3.

Estimation of the atomic fraction of cationic and neutral Pt from IRAS spectra.

To estimate the number of neutral and cationic Pt atoms, coverages of CO molecules adsorbed on cationic ($\theta_{CO,\,C}$) and neutral ($\theta_{CO,\,N}$) Pt atoms were estimated using Supplementary Equations 2 and 3,

$$\theta_{CO,C} = I_C \, / \, \alpha_C \qquad (2)$$

$$\theta_{CO,N} = I_N \, / \, \alpha_N \qquad (3),$$

where I_C and I_N are peak intensities of IRAS spectra at 2044 cm^{-1} and 2020 cm^{-1}, respectively (**Fig. 3a**), and α_C and α_N are the extinction coefficients of the adsorbed CO molecules on cationic and neutral Pt atoms, respectively. To obtain $\theta_{CO,\,C}$ and $\theta_{CO,\,N}$ from the peak intensity, the extinction coefficients were estimated as explained below. Total coverage of adsorbed CO (θ_{CO}) on Pt_n is $\theta_{CO,C} + \theta_{CO,N}$, and thus

$$\theta_{CO} = I_C/\alpha_C + I_N/\alpha_N \qquad (4).$$

Assuming that α_C and α_N are independent of the size of Pt cluster and adsorbed CO coverage, we can set up simultaneous equations by substituting experimental values for θ_{CO}, I_C, and I_N at each cluster size, where θ_{CO} was obtained using TPD

(**Supplementary Fig. 11a**). By solving the simultaneous equation (4) for n = 19 and 24, α_C and α_N were obtained. To confirm the accuracy of the obtained extinction coefficients, θ_{CO} calculated using the extinction coefficients and IRAS peak intensities was compared with that obtained by TPD (**Supplementary Fig. 11b**). The correlation between the θ_{CO} calculated from IRAS intensity and that obtained by TPD is good (R^2 = 0.87), indicating that CO coverages can be accurately estimated from IRAS peak intensity. Therefore, we can estimate $\theta_{CO, C}$ and $\theta_{CO, N}$ using the IRAS peak intensities.

The fractions of cationic and neutral Pt atoms (**Fig. 3b**) were calculated as $\theta_{CO,C}/(\theta_{CO,C} + \theta_{CO,N})$, and $\theta_{CO,N}/(\theta_{CO,C} + \theta_{CO,N})$, respectively. The ratio of neutral to cationic Pt atoms (**Fig. 3g**) was given by $\theta_{CO,N}/\theta_{CO,C}$. Although the IRAS peak shows a slight blue shift with increasing coverage (**Supplementary Fig. 11c**) due to intermolecular interactions like dipole-dipole coupling [8], the linear correlation between the estimated coverages by IRAS and TPD indicates that the intermolecular interactions are negligible for the coverage estimation.

Supplementary Note 4.
DFT calculation of Pt clusters on Al_2O_3

The total binding energy of each Pt cluster on Al_2O_3 was determined by subtracting the total energy of Al_2O_3 and Pt from the total energy of Pt cluster adsorbed Al_2O_3 system using DFT calculations. We calculated the total binding energy of single-layer Pt clusters with different numbers of Pt atoms in the unit cell and configurations (**Supplementary Fig. 12**). In this calculation, all Pt atoms were directly coordinated with Al_2O_3 surface atoms. The total binding energy divided by the number of Pt atoms equals to the averaged bond energy of individual bonding of Pt to Al_2O_3 (E_b), as shown in Supplementary Equation 5:

$$E_b = [E(\mathrm{Pt}_{n'}/\mathrm{Al_2O_3}) - (E(\mathrm{Al_2O_3}) + E(\mathrm{Pt}_{n'})]/n' \quad (5)$$

where $E(\mathrm{Pt}_{n'}/\mathrm{Al_2O_3})$, $E(\mathrm{Al_2O_3})$, and $E(\mathrm{Pt}_{n'})$ are the total energies of the $\mathrm{Pt}_{n'}/\mathrm{Al_2O_3}$ slab, Al_2O_3 slab, and $\mathrm{Pt}_{n'}$ cluster, and n' is the number of Pt atoms in each unit cell. **Figure 3d** shows the average bond energy of Pt binding to Al_2O_3 as a function of the average coordination number of Pt to neighbouring Pt atoms, calculated using the slab model (**Supplementary Fig. 12a-c**).

Vila et al. have demonstrated the charge transfer from Pt to an Al_2O_3 support[9]. We have also achieved similar results, as summarized in Supplementary Fig. 12d.

Supplementary Note 5.

Simulation of cluster morphology using bond additivity model.

The size-dependency of Pt_n morphology on Al_2O_3 (**Fig. 2a**) was analysed by calculating the binding energy (E_{bd}) of model Pt_n clusters using the bond additivity model (BAM), which provides E_{bd} as a sum of Pt–Pt and Pt–support bond energy (equation (3) of the main text). By comparing the calculated E_{bd} of single-to-quadruple layer model structures (**Fig. 4b**), most stable morphology was assigned for each cluster size.

Since Pt–Al_2O_3 bond energy increases linearly with decreasing $\mathrm{CN}_{\mathrm{Pt–Pt}}$, as revealed by DFT calculation (**Fig. 3d**), we modelled the dependency of the Pt–Al_2O_3 bond energy on $\mathrm{CN}_{\mathrm{Pt–Pt}}$ as

$$E_{\mathrm{Pt\text{-}support},\,j} = \mathrm{a} \times \mathrm{CN}_{\mathrm{Pt–Pt},\,j} + \mathrm{b} : \quad \mathrm{CN}_{\mathrm{Pt–Pt},\,j} \le 5 \quad (6)$$

$$E_{\mathrm{Pt\text{-}support},\,j} = E_{\mathrm{Pt\text{-}support}}\ (\mathrm{CN}_{\mathrm{Pt–Pt}} = 5): \quad \mathrm{CN}_{\mathrm{Pt–Pt},\,j} \ge 6 \quad (7),$$

where $E_{\mathrm{Pt\text{-}support},\,j}$ is the bond energy of jth Pt–support bond, $\mathrm{CN}_{\mathrm{Pt–Pt},\,j}$ is the $\mathrm{CN}_{\mathrm{Pt–Pt}}$ of

the Pt atom with the *j*th Pt–support bond, and a and b are constants. Although **Fig. 3d** shows the relationship between the average Pt–support bond energy (⟨$E_{\text{Pt-S}}$⟩) and the average $CN_{\text{Pt–Pt}}$ (⟨$CN_{\text{Pt–Pt}}$⟩), the linear relation would also hold for each Pt–Al_2O_3 bond because the ⟨$E_{\text{Pt-support}}$⟩ of Pt_1, Pt_2, Pt_4, and Pt_8 on Al_2O_3, for which ⟨$CN_{\text{Pt–Pt}}$⟩ equals to $CN_{\text{Pt–Pt},\,j}$, shows the same linear $CN_{\text{Pt–Pt}}$ dependency with that of Pt_3, Pt_5, Pt_6, and Pt_7 (**Fig. 3d**).

To calculate $CN_{\text{Pt–Pt}}$-dependent Pt–support bond energy, two appropriate bond energies for $CN_{\text{Pt–Pt}}$ = 0 and 5 were assigned, as schematically illustrated in **Fig. 4c**, and the slope of the function and the intercept of Supplementary Equation 6 were calculated. Then the coordination number of the Pt atom of interest in the cluster is inserted in this equation to obtain the bond energy of that Pt atom. We repeated this process for all other Pt atoms in the cluster by assigning the different bond energies at $CN_{\text{Pt–Pt}}$ = 0 and 5 (**Supplementary Fig. 13a**) until the single-to-double and double-to-triple layer cluster transitions appeared at n = 18–19 and at n = 24–30 (**Fig. 4d** and **Supplementary Fig. 13b**), respectively, as observed using STM (**Fig. 2a**). **Supplementary Figure 13b** shows the calculated E_{bd} (in the units of eV/atom), which were fitted by a function of $n^{-1/3}$ (refs. [10,11]), and the cross point between the fitted curves is defined as the morphological transition size. For the Pt–Pt bond energy, the bulk value of 0.98 eV is used. Note that the Pt–Pt bond energy also depends on the Pt–Pt coordination number (**Supplementary Fig. 15** and **Supplementary Note 6**). However, the effect of this dependency on cluster morphology is negligible because the morphological transition observed using STM can be reproduced by BAM with only the $CN_{\text{Pt–Pt}}$ dependency of Pt–Al_2O_3 bond energy.

We confirmed that BAM with constant Pt–Pt and Pt–support bond energies does not

reproduce the appearance of triple layer at $n \geq 30$, as observed using STM. **Supplementary Figure 14a** shows the E_{bd} calculated using constant bond energies of Pt–Pt (0.98 eV) and Pt–Al_2O_3 (2 eV), showing that the most stable morphology for $n \leq 18$ is single layer, and for $19 \leq n \leq 40$ is double layer. **Supplementary Figures 13b-d** show how the most stable morphology depends on size and bond energy. For example, **Supplementary Figure 13b** shows the most stable morphology as a function of the Pt–Al_2O_3 bond energy and size, when the Pt–Pt bond energy is 0.5 eV, implying that morphological transition from single to double layer occurs at n = 18–19, when the Pt–Al_2O_3 bond energy is 1.0 eV, but that from double to triple layer does not occur at $n \leq 40$. **Supplementary Figs. 13c,d** also show the most stable morphologies for Pt–Pt bond energies of 0.98 and 2 eV, where the transition from the single to double and double to triple layer cluster do not appear as observed using STM (**Fig. 2a**). Therefore, the BAM with constant bond energy is insufficient to explain the size-dependency of cluster morphology.

Supplementary Note 6.

DFT calculation of Pt–Pt bond energy for freestanding Pt cluster.

The relationship between the Pt–Pt bond energy ($E_{\text{Pt-Pt}}$) and Pt–Pt coordination number ($\text{CN}_{\text{Pt–Pt}}$) was investigated using DFT calculations. To obtain the on $\text{CN}_{\text{Pt–Pt}}$ dependency of $E_{\text{Pt-Pt}}$, the cohesive energy of freestanding Pt_n clusters (E_{coh}) was calculated by

$$E_{\text{coh}} = [E(\text{Pt}_n) - n \times E(\text{Pt})]/n \qquad (8)$$

where $E(\text{Pt}_n)$ and $E(\text{Pt})$ are the total energies of the Pt_n cluster and Pt atom, respectively[12] (**Supplementary Fig. 15a**). Note that a Pt–Pt bond connects two Pt atoms, and its bond energy would depend on $\text{CN}_{\text{Pt–Pt}}$ of both atoms. We labelled a Pt–Pt bond

by the sum of $CN_{Pt–Pt}$ of both atoms (SCN). For example, Pt_4 tetrahedron has six Pt–Pt bonds and the $CN_{Pt–Pt}$ of all Pt atoms is three, and therefore SCN of each bond is six (**Supplementary Fig. 15b**). By counting the number of Pt–Pt bonds and the SCN for the freestanding Pt_n clusters, the average SCN (⟨SCN⟩) was calculated by

$$\langle \mathrm{SCN} \rangle = \sum_{i=1}^{N_{\mathrm{Pt-Pt}}} \mathrm{SCN}_i \Big/ N_{\mathrm{Pt-Pt}} \quad (9),$$

where SCN_i is SCN of *i*th Pt–Pt bond, and N_{Pt-Pt} is the number of Pt–Pt bond. ⟨SCN⟩ was calculated for each cluster, and the cluster size *n* was converted to the average SCN, as shown in **Supplementary Fig. 15c**. The relationship between the Pt–Pt bond energy and the SCN was obtained by converting the E_{coh} to average E_{Pt-Pt} ($\langle E_{Pt-Pt} \rangle = E_{coh} / N_{Pt-Pt}$), and $\langle E_{Pt-Pt} \rangle$ was found to correlate very well with ⟨SCN⟩ as

$$\langle E_{\mathrm{Pt-Pt}} \rangle = \mathrm{c} \, \langle \mathrm{SCN} \rangle^{-0.5} + \mathrm{d} \quad (10),$$

where c and d are constants, as shown as solid lines in **Supplementary Fig. 15d**.

Supplementary Note 7.

Estimation of the peak integral for TPR measurements.

To estimate the area below a peak, the background must be subtracted from the measured data. When we draw a background line, we should be careful not to draw the line through data points where some reactions are taking place. Thus, we collected ≥10 data points from 100 K and below and from 500 K and above, as schematically illustrated in **Supplementary Fig. 17a**, to draw the background line between 100 to 500 K, where CO oxidation occurs. Then, we subtracted the background from the obtained spectra. The error bars in **Figs. 1b**, **Fig. 3g**, and **Fig. 5b** for Pt_{30}/Al_2O_3 are the standard deviations estimated from the calculated area of the peak in each spectrum from the

TPR experiments, which were performed in triplicate.

Supplementary Note 8.

Cluster height estimation by STM.

Supplementary Fig. 7b schematically illustrates the STM line profile for the surface of Pt_n/Al_2O_3/NiAl. The thickness of the Al_2O_3 layer is 0.54 nm (ref. [13]), but at the scan voltage of 3.5 V at a constant current of 0.1 nA, the apparent STM height was 0.33 nm, as represented by the blue line; thus, the height of Al_2O_3 is underestimated. This can be understood by the band diagram shown in **Supplementary Fig. 7c**, which demonstrates that the electrons (corresponding to a 0.1 nA current) are tunnelling not only to the density of states of Al_2O_3 but also to those of NiAl. As the STM tip continues scanning the surface of Al_2O_3 from left to right, it encounters the Pt atom, and the tip moves up and provides information of the height of the Pt atom, as illustrated in **Supplementary Fig. 7b**. The apparent height was measured to be about 0.4 nm. Nevertheless, the apparent height of Pt is actually affected by the underestimation of the Al_2O_3 thickness described above, and thus, the Pt height is overestimated by about 0.21 nm (which is calculated from the real height of Al_2O_3, i.e. 0.54 nm - 0.33 nm), as depicted in **Supplementary Fig. 7b**. Hence, the height of the Pt should be assigned to 0.19 nm (= 0.4 nm - 0.21 nm), which is in good agreement with the Pt(111) height of 0.23 nm. Thus, we assigned the clusters with an average height of around 0.4 nm as a single-layer Pt cluster. For the second and third layers, the apparent height was 0.14 and 0.12 nm, respectively. These heights are slightly thinner than the atomic step of Pt(111), but we assigned them as the second and third layers since such a height decrease is probable if the first layer is slightly relaxed compared to the closely packed Pt(111) atomic

arrangement. Thus, the second layer atoms may be sunken into the first layer, which could also happen for the third layer. High-resolution STM images can be acquired under different conditions, as shown in **Supplementary Fig. 7d-e**, but such conditions break the Pt cluster. Accordingly, we performed STM under conditions that do not alter the Pt cluster during scanning. We also note that the heights in all STM images were calibrated with respect to a single atomic step on NiAl for all the imaging conditions.

Supplementary Figures

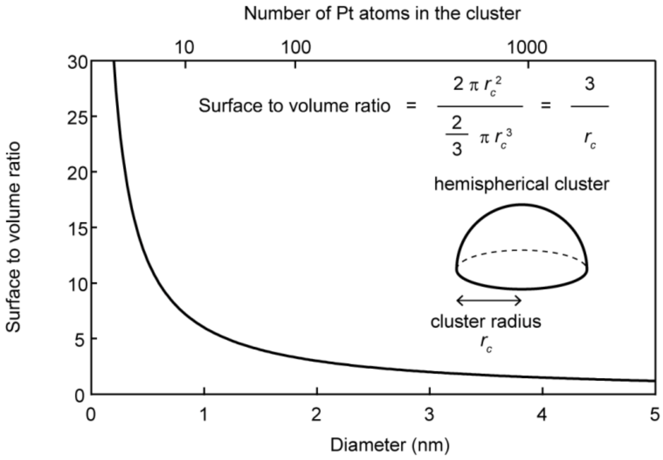

Supplementary Fig. 1 | Calculated fraction of surface-exposed Pt atoms in a hemispherical cluster. Model calculation of surface-to-volume ratio for a hemispherical cluster. This ratio monotonically increases with decreasing cluster diameter. The number of Pt atoms in the cluster was calculated by multiplying the volume of the hemispherical nanoparticle and the volume density of bulk Pt (66.2 atoms nm^{-3}).

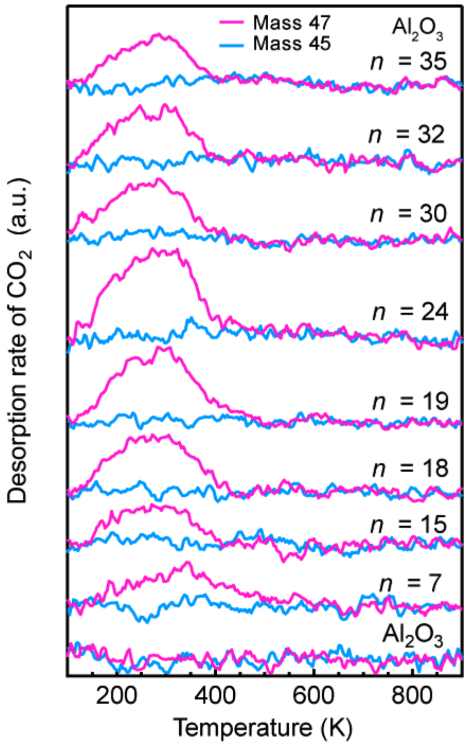

Supplementary Fig. 2 | TPR spectra of CO_2 measured using masses 47 ($^{13}CO^{18}O$) and 45 ($^{13}CO_2$) for Pt_n/Al_2O_3. The Pt_n-deposited surfaces were exposed to 1000 L of $^{18}O_2$ at 300 K followed by saturation adsorption of ^{13}CO at 88 K, and then, TPR measurements were performed. Desorption signal of $^{13}CO_2$ (mass 45) from Pt_n/Al_2O_3 was below the detection limit; thus, the CO_2 is formed through the reaction between the introduced ^{13}CO and ^{18}O ($^{13}CO + {}^{18}O \rightarrow {}^{13}CO^{18}O$; mass 47).

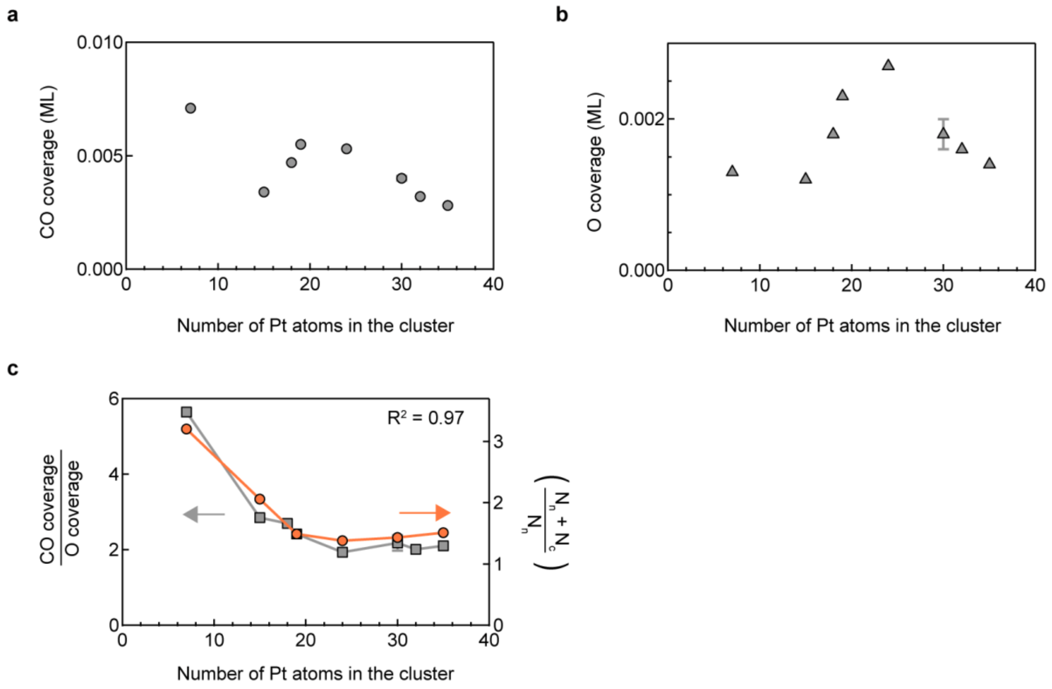

Supplementary Fig. 3 | CO and O coverages adsorbed on mass-selected Pt_n clusters on Al_2O_3. a, CO and O (**b**) coverages. The Pt_n deposited surfaces were exposed to 1000 L of $^{18}O_2$ at 300 K to saturate the Pt_n clusters with ^{18}O atoms, followed by saturation adsorption of ^{13}CO at 88 K, and finally, the TPR measurement was performed. Pt coverage was 0.02 ML (1 ML = 1.5×10^{15} atoms/cm^2). CO coverage (**a**) was estimated as the sum of the amount of produced CO_2 (Fig. 1b) and of unreacted CO (Fig. 1d). We detect no O_2 desorption during TPR as same as the previous report. Thus O coverage (**b**) was calculated by assuming that all the adsorbed O is consumed by CO during the reaction based on the previous report[14]. **c**, Ratio of CO coverage to O coverage. This ratio correlates well with $(N_n+N_c)/N_n$, suggesting that cationic Pt has a low oxygen affinity. $(N_n+N_c)/N_n$ was calculated from the IRAS results (Fig. 3). The error bars are standard deviation.

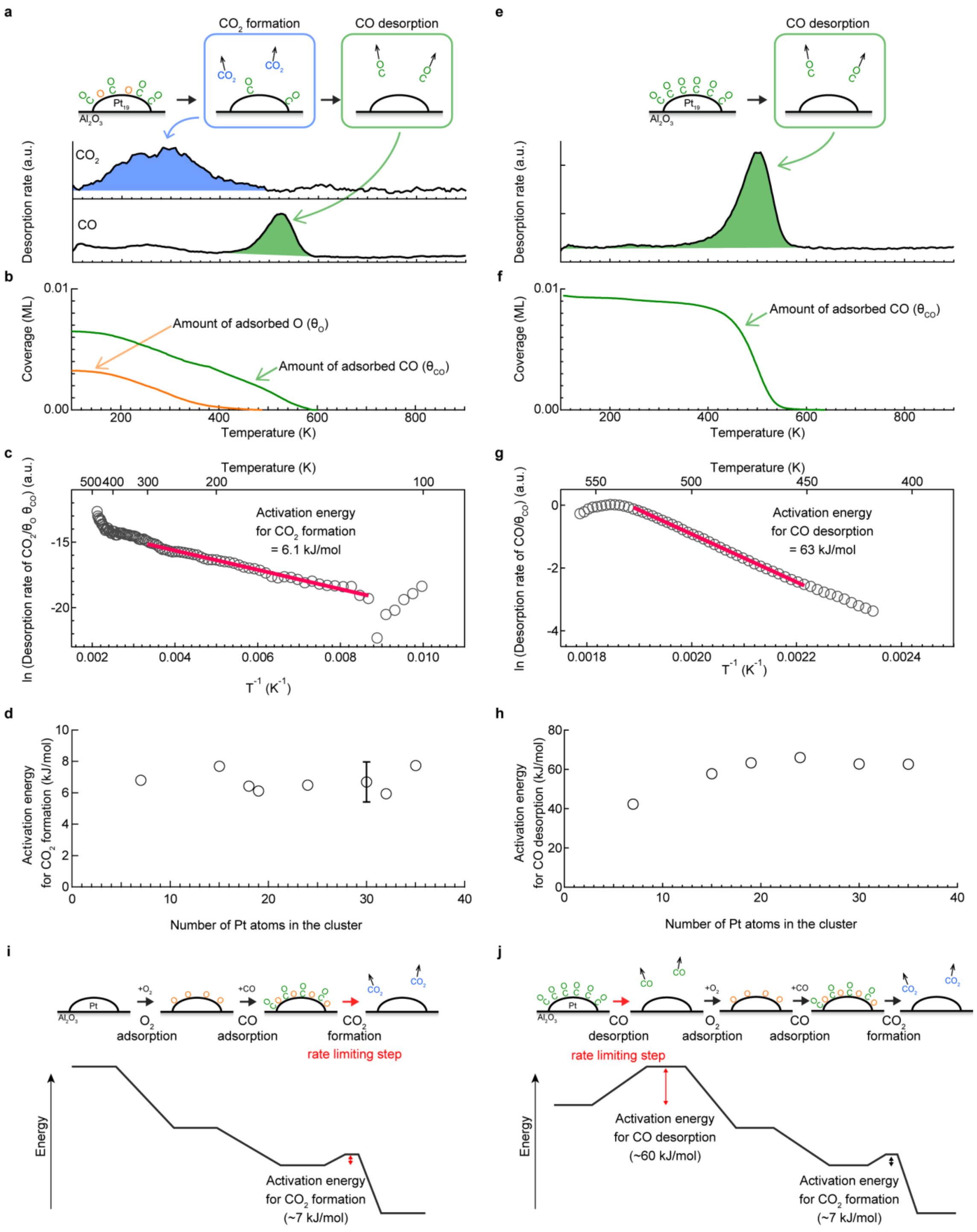

Supplementary Fig. 4 | Activation energies for CO_2 formation and CO desorption over Pt_n/Al_2O_3. a, CO_2 (m/z = 47) and CO (m/z = 29) TPR spectra over Pt_{19}/Al_2O_3. The Pt_{19} deposited surface was exposed to 1000 L of $^{18}O_2$ at 300 K to saturate the Pt_{19} clusters by ^{18}O atoms, followed by saturation adsorption of ^{13}CO at 88 K, and finally,

the TPR measurement was performed. **b**, Amounts of adsorbed ^{13}CO and ^{18}O over Pt_{19}/Al_2O_3 as a function of temperature. To estimate these amounts, the number of produced CO_2 molecules and unreacted CO molecules were determined from the TPR peak areas. The amount of adsorbed CO was determined by summing those of produced CO_2 and unreacted CO. The amount of adsorbed O was determined to be equal to that of produced CO_2. **c**, Coverage-corrected Arrhenius plot for CO_2 formation over Pt_{19}/Al_2O_3. The activation energy was determined from the slope of the coverage-corrected Arrhenius plot[3,4] (natural logarithm of $(r_{CO_2}/(\theta_{CO}\times\theta_{O}))$ versus reciprocal temperature, where r_{CO_2} represents the desorption rate of CO_2, and θ_{CO} and θ_{O} represent the amounts of adsorbed CO and O, respectively). **d**, Size dependency of the activation energy for CO_2 formation. The error bar was determined from multiple measurements on a single cluster size. **e**, CO (m/z = 29) TPD spectrum over Pt_{19}/Al_2O_3. The Pt_{19} deposited surface was saturated by ^{13}CO at 88 K and then the TPD measurement was performed. **f**, Amount of adsorbed ^{13}CO over Pt_{19}/Al_2O_3 as a function of temperature. **g**, Coverage-corrected Arrhenius plot for CO desorption over Pt_{19}/Al_2O_3. The activation energy was determined from the slope of the coverage-corrected Arrhenius plot (natural logarithm of (r_{CO}/θ_{CO}) versus reciprocal temperature, where r_{CO} and θ_{CO} represent the desorption rate of CO and the amount of adsorbed CO, respectively). **h**, Size dependency of the activation energy for CO desorption. **i**,**j**, Representative potential energy diagrams for CO oxidation over an oxygen-saturated Pt_n cluster (**i**) and a CO-saturated Pt_n cluster (**j**). The error bar in (d) is standard deviation.

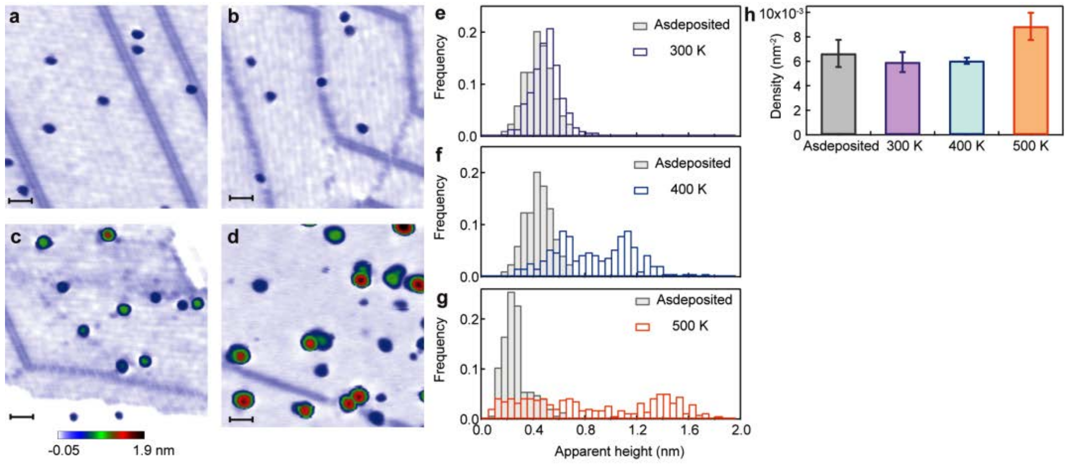

Supplementary Fig. 5 | STM images of pristine and annealed O_2-exposed Pt_{15}/Al_2O_3 surface and the cluster height distributions. a,**b**, STM images of pristine Pt_{15}/Al_2O_3 (**a**), and after the exposure to 1000 L O_2 at 300 K (**b**), 400 K (**c**), and 500 K (**d**). **e-g,** Histograms of cluster height before and after O_2 exposure at 300 K (**e**), 400 K (**f**), and 500 K (**g**). Cluster height increased after O_2 exposure above 400 K, indicating that the morphology changed upon O adsorption above 400 K. **h**, Cluster density before and after O_2 exposure. The increase in cluster density for O_2-exposed surface at 500 K indicates that Pt_{15} clusters are decomposed and aggregated by this treatment. Scale bars in (**a**)-(**d**) correspond to 5 nm. [(a)-(c), (e), (f): V_s = 3.5 V and I_t = 0.1 nA; (d),(g): V_s = 4.5 V and I_t = 5 pA]. For each STM condition, the height was calibrated with respect to a single atomic step on NiAl. The error bars in (h) is standard deviation.

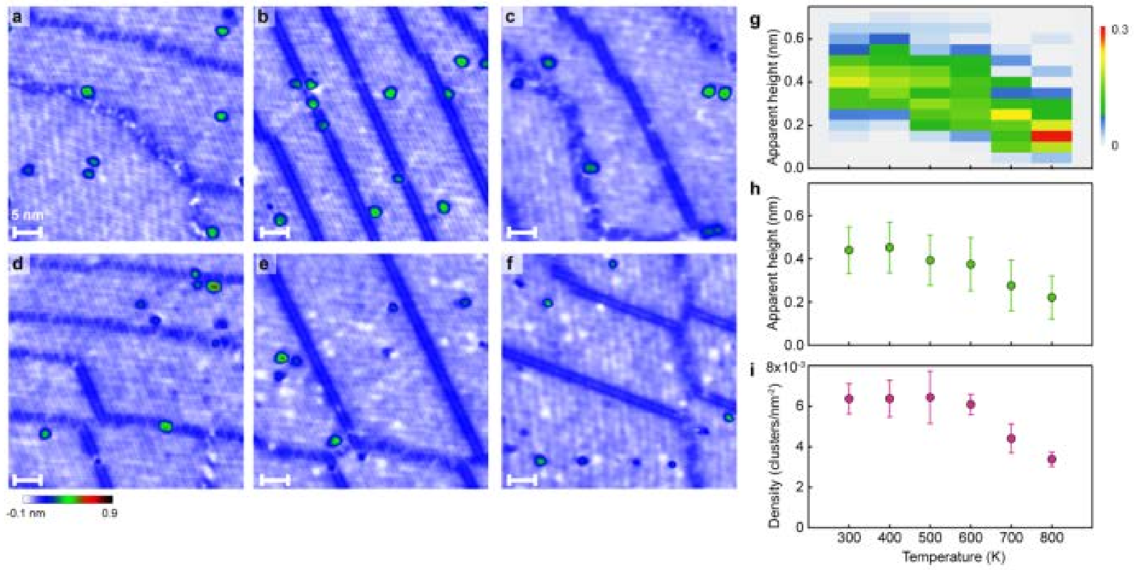

Supplementary Fig. 6 | STM images of pristine and annealed Pt_{15}/Al_2O_3 surface and their cluster height distributions and densities. a-f, STM images of pristine (**a**) and Pt_{15}/Al_2O_3 annealed at 400 K (**b**), 500 K (**c**), 600 K (**d**), 700 K (**e**), and 800 K (**f**). **g-i**, Height distribution histogram (**g**), average heights (**h**), and cluster density (**i**). Scale bars in (**a**)-(**f**) are 5 nm. [V_s = 3.5 V and I_t = 0.1 nA]. The cluster height and density are constant at temperatures up to 600 K, indicating that the Pt_{15} clusters are thermally stable up to 600 K. The error bars in (h) and (i) are standard deviation.

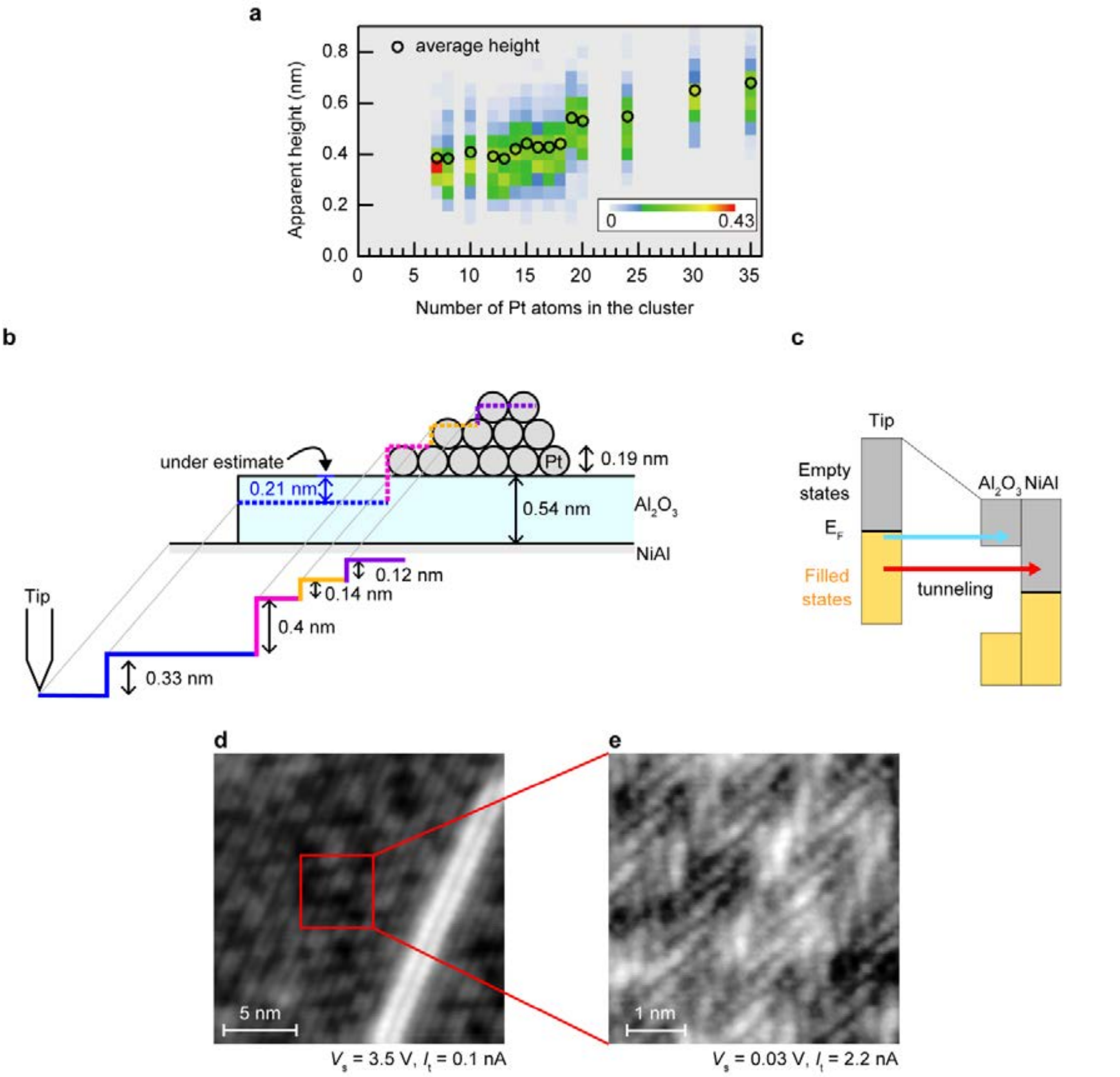

Supplementary Fig. 7 | Height distribution of the Pt clusters for each cluster size. a, Histogram of apparent height of Pt_n clusters on Al_2O_3. Average heights of Pt clusters in **Fig. 2a** were estimated from this data. **b**, Schematic illustration of the STM line profile for the model $Pt/Al_2O_3/NiAl$ structure. The thickness of the Al_2O_3 film was underestimated by 0.21 nm at a positive sample bias of 3.5 V and a tunnelling current of 0.1 nA. **c**, Schematic illustration of electron tunnelling from the STM tip to Al_2O_3 and the NiAl substrate. **d**, STM image of the Al_2O_3 surface measured with V_s = 3.5 V and I_t = 0.1 nA. **e**, Atomic-resolution STM image of the Al_2O_3 surface measured with V_s = 0.03 V and I_t = 2.2 nA.

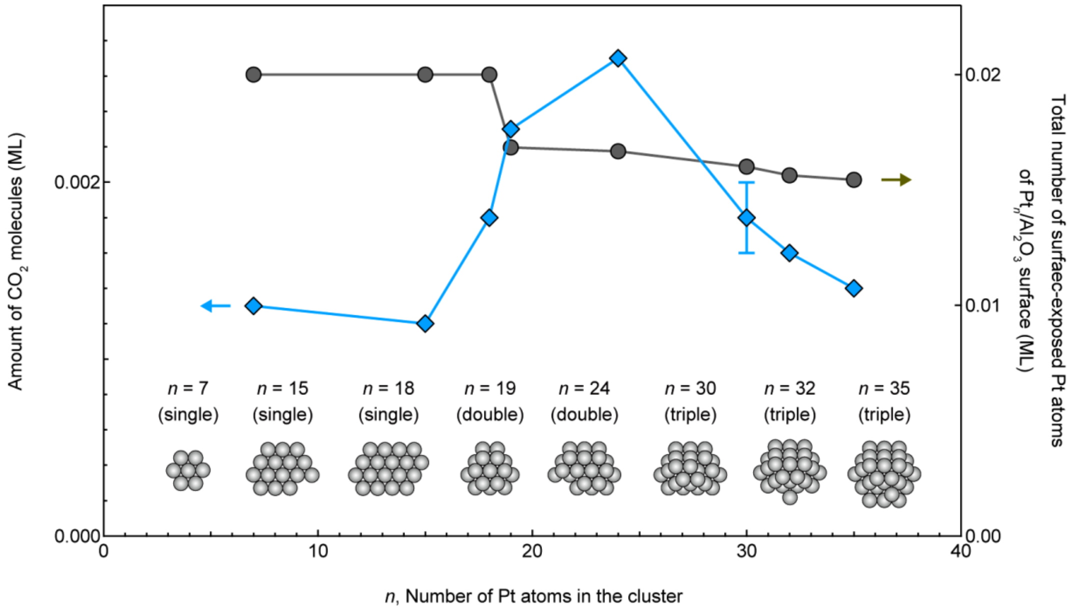

Supplementary Fig. 8 | Relationship between the amount of produced CO_2 and that total number of surface-exposed Pt atoms of Pt_n/Al_2O_3 surface. Structures of Pt clusters of different sizes are shown in insets, which are modelled based on the STM observations (**Fig. 2**). Total number of surface-exposed Pt atoms was counted based on these structures. There was no peak in the total number of surface-exposed Pt atoms along with the cluster size, and thus, no correlation to the amount of produced CO_2 molecules. The Pt coverage was 0.02 ML. The error bar is standard deviation.

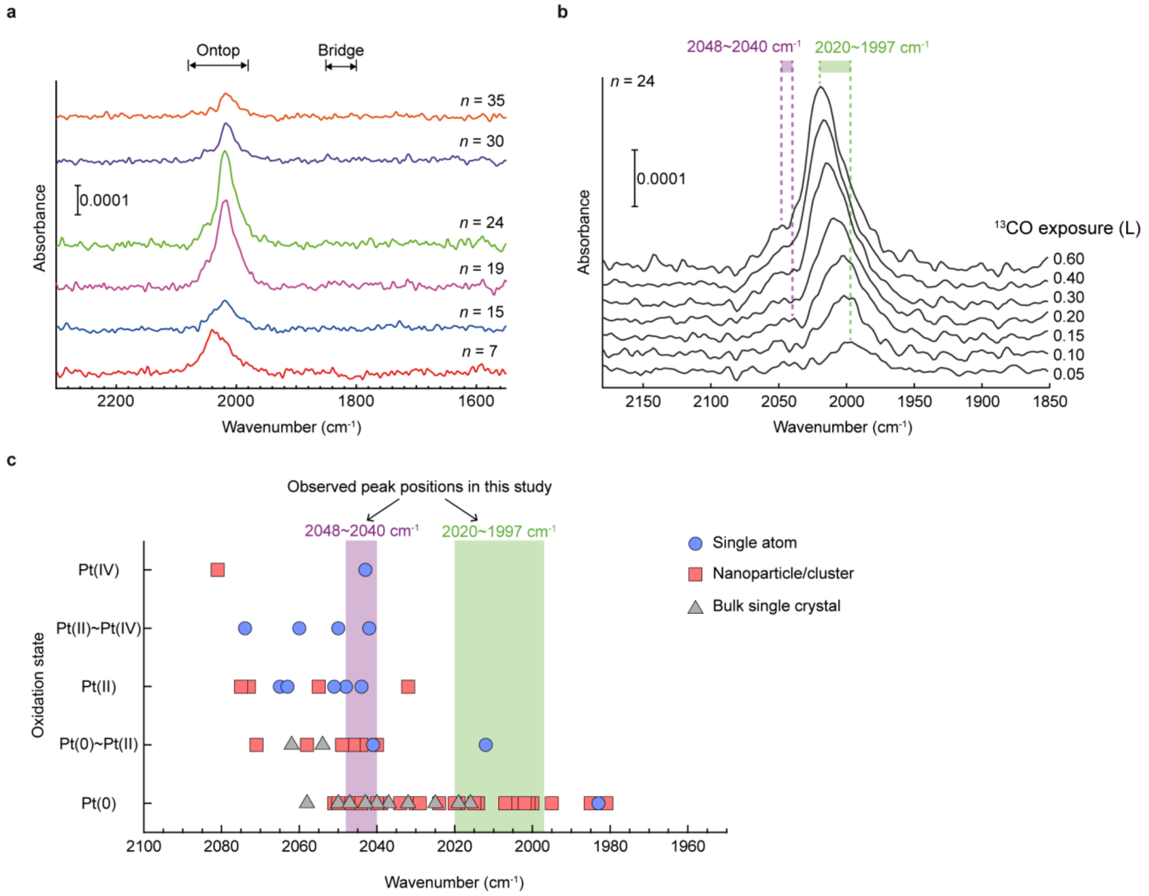

Supplementary Fig. 9 | IRAS spectra of ^{13}CO adsorbed on Pt$_n$/Al$_2$O$_3$ and reported vibrational frequencies of ontop ^{13}CO on Pt catalysts. a, IRAS spectra of ^{13}CO adsorbed on Pt$_n$/Al$_2$O$_3$. The Pt$_n$ deposited surfaces were saturated by ^{13}CO at 88 K, and IRAS measurements were performed. CO molecules adsorb only on ontop sites of Pt clusters. **b**, IRAS spectra of ^{13}CO adsorbed on Pt$_{24}$/Al$_2$O$_3$ as a function of ^{13}CO exposure. The Pt$_{24}$ deposited surface was exposed to ^{13}CO at 88 K, and then IRAS measurements were performed. The observed frequency ranges of the two peaks are shown in green (2,020-1,997 cm^{-1}) and purple (2,048-2,040 cm^{-1}) bars respectively. **c**, Reported vibrational frequencies of ontop adsorbed ^{13}CO on Pt catalysts. Wavenumbers plotted in the figure and corresponding references are summarized in **Supplementary Table 1**.

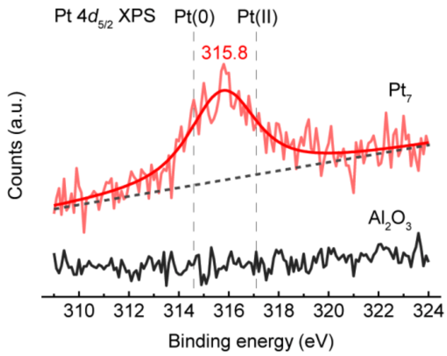

Supplementary Fig. 10 | XPS spectrum of Pt_7 on Al_2O_3. XPS spectrum of the Pt $4d_{5/2}$ region for Pt_7/Al_2O_3 (red) and the Al_2O_3 substrate (black). XPS data showing the Pt $4d_{5/2}$ peak with literature values for Pt(0) and Pt(II) (ref. [15]) displayed for reference. The Pt $4d_{5/2}$ XPS spectrum was fitted using a Voigt function centred at 315.8 eV (red solid curve). XPS spectra were recorded at 300 K.

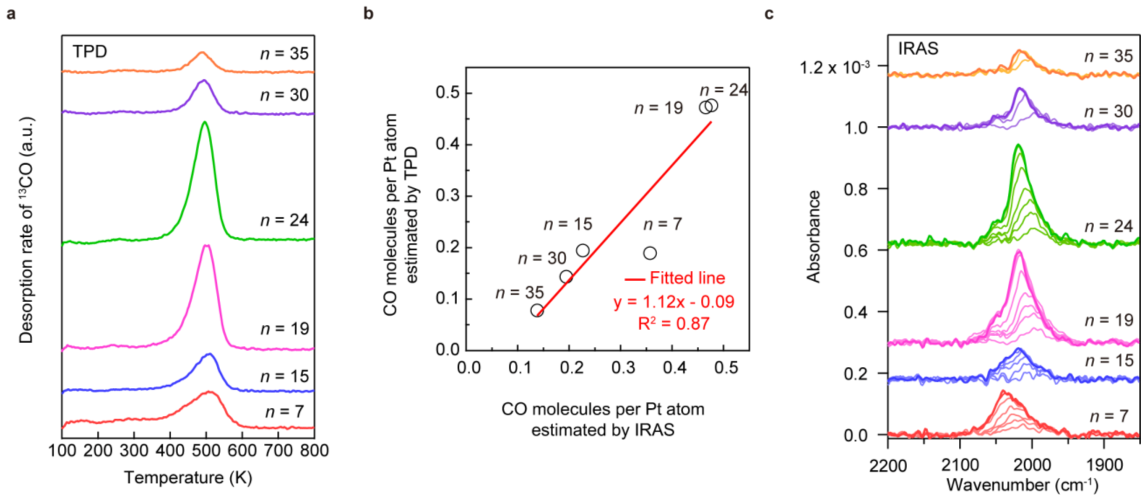

Supplementary Fig. 11 | CO adsorption onto mass-selected Pt_n clusters on Al_2O_3.

a, Temperature-programmed desorption (TPD) spectra (m/z = 29) of ^{13}CO over Pt_n/Al_2O_3. The Pt_n/Al_2O_3 surface was exposed to ^{13}CO at 88 K, followed by TPD measurement. **b,** Relationship between the amount of adsorbed CO estimated by TPD and that by IRAS. The fitted line shows a unit slope, and the y-intercept is almost zero, implying that IRAS can also be used to estimate the CO coverage, as discussed in **Supplementary Note 3**. **c**, IRAS spectra of ^{13}CO adsorbed on Pt_n/Al_2O_3 as a function of ^{13}CO exposure. The Pt_n/Al_2O_3 surface was exposed to ^{13}CO at 88 K, followed by IRAS measurement at 88 K.

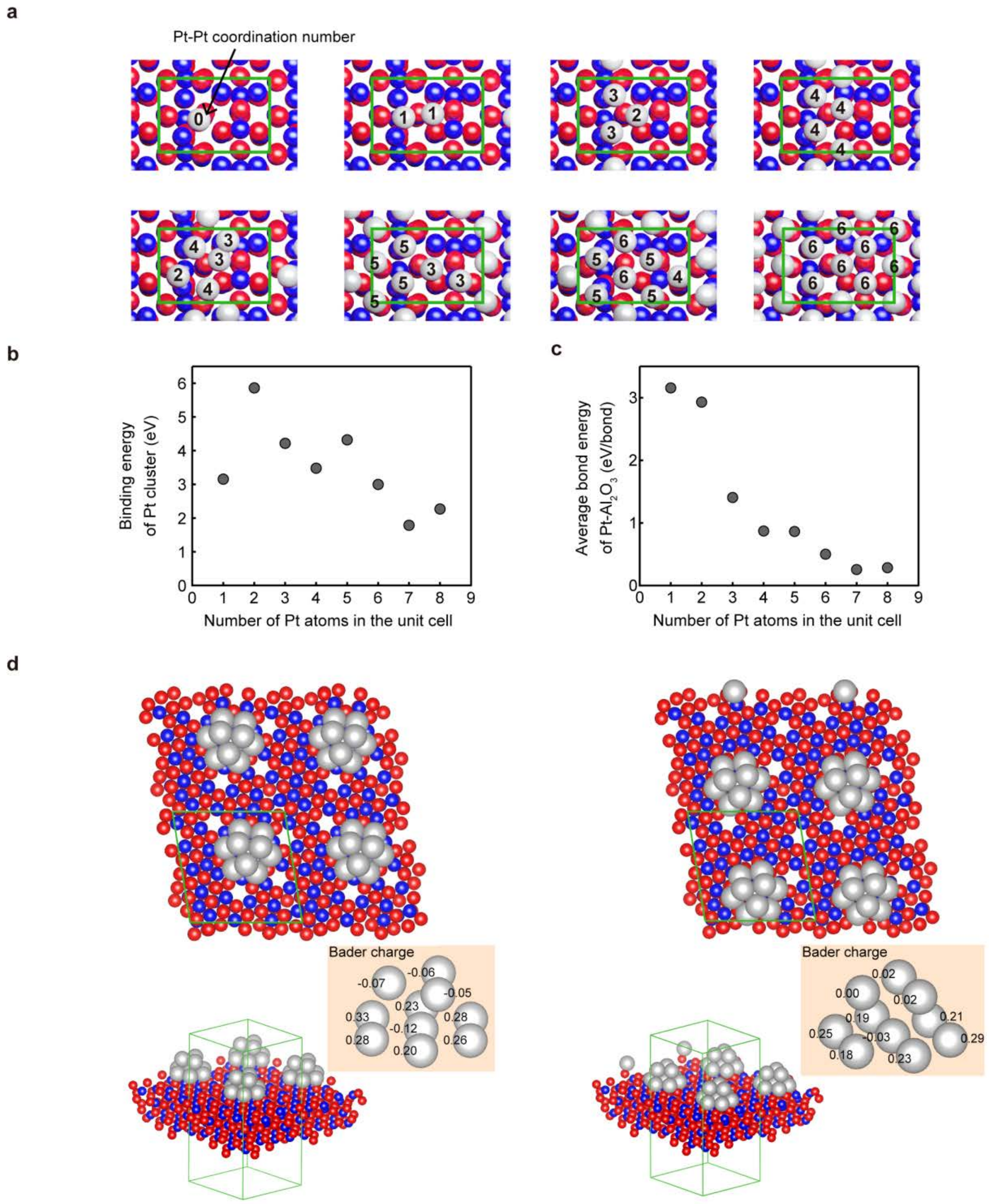

Supplementary Fig. 12 | Average bond energy of Pt–Al_2O_3 obtained using DFT calculations. a, Pt/Al_2O_3 configurations used to obtain the binding energies of Pt clusters via DFT calculations. The solid rectangle (green) represents the unit cell, while the blue, red, and grey spheres represent Al, O, and Pt atoms, respectively. The integral

numbers on the Pt atoms represent the Pt–Pt coordination numbers, which were used to calculate the average coordination number of the Pt atoms in the unit cell. **b**, Calculated binding energy of Pt clusters as a function of the number of Pt atoms in the unit cell. **c**, Calculated average bond energy of Pt–Al_2O_3 as a function of the number of Pt atoms in the unit cell, in which the average bond energy was calculated by dividing the binding energy of the Pt cluster by the number of Pt atoms in the unit cell. **d**, Structural models of Pt_{10} cluster adsorbed on the Al_2O_3 surface constructed based on the ref. 9 to calculate Bader charges of the Pt atoms, demonstrating charge transfer from Pt to Al_2O_3 and existence of oxidized or cationic Pt atoms.

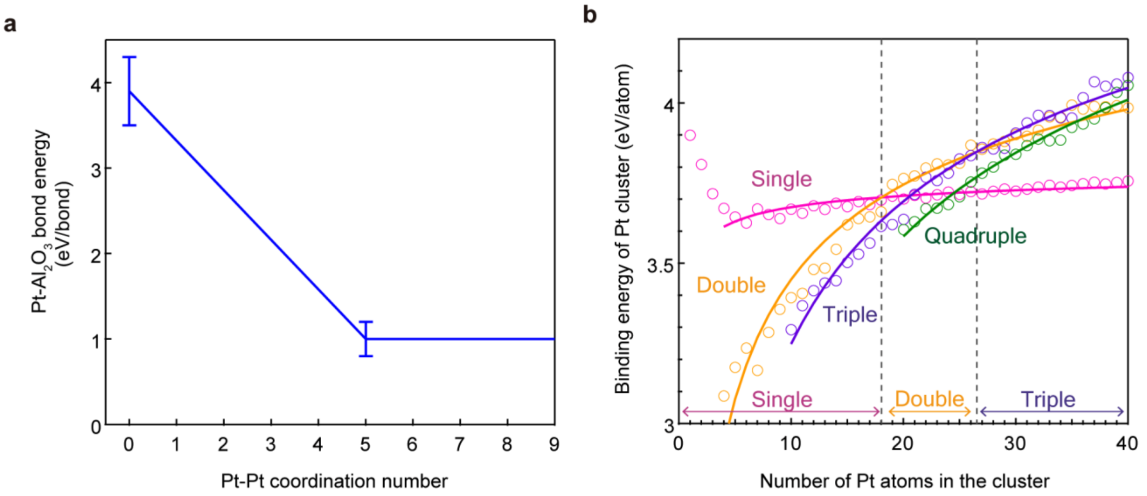

Supplementary Fig. 13 | Simulation of the cluster morphology of Pt_n/Al_2O_3 using our bond additivity model with coordination-number-dependent Pt–Al_2O_3 bond energy and bulk Pt–Pt bond energy. a, Simulated dependency of Pt–Al_2O_3 bond energy on the coordination number, where the single-to-double and double-to-triple layer cluster transitions appeared at n = 18–19 and at n = 24–30 (**Fig. 4d**), respectively, as observed using STM (**Fig. 2a**). Error bars represent the upper and lower limits of the bond energy. **b**, Simulated binding energy of the model Pt_n clusters using a Pt–Al_2O_3 bond energy of 3.9 eV at $CN_{Pt–Pt}$ = 0 and 1.0 eV at $CN_{Pt–Pt}$ = 5. Calculated adsorption energies for single (pink), double (orange), triple (purple), and quadruple (green) Pt_n clusters were fitted by A $n^{-1/3}$ + B (A and B are constant) to obtain the intersection point of the fitted lines, which corresponds to the morphological transition size. Vertical dashed lines show the simulated morphological transition sizes from single to double (n = 18–19) and double to triple layer (n = 26–27).

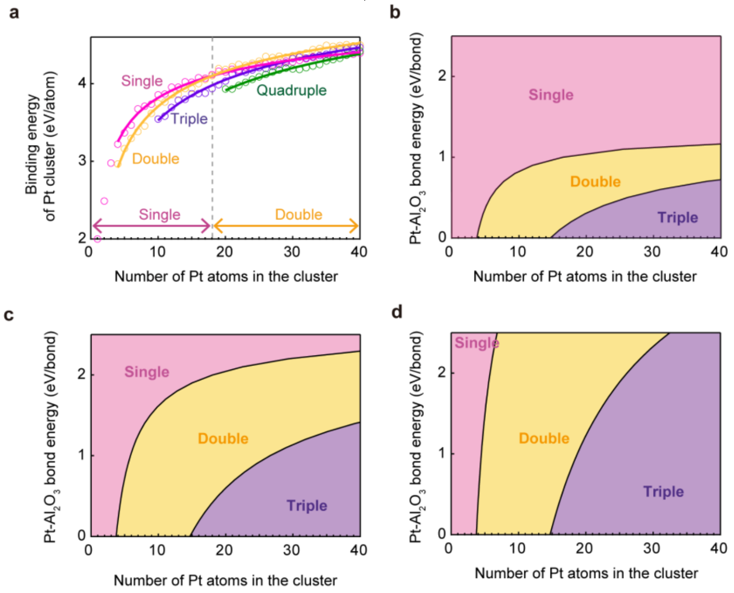

Supplementary Fig. 14 | Simulation of cluster morphology for Pt_n/Al_2O_3 using previously proposed bond additivity model with constant Pt–Al_2O_3 and Pt–Pt bond energies. a, Calculated binding energy of the single (pink), double (orange), triple (purple), and quadruple (green) model Pt_n clusters (**Fig. 4b**) using the bond additivity model (BAM), with constant bond energies of Pt–Pt (bulk value = 0.98 eV) and Pt–Al_2O_3 (2 eV). Calculated binding energies were fitted by A $n^{-1/3}$ + B (A and B are constant) to obtain the intersection point of the fitted lines, corresponding to the morphological transition size. Vertical dashed line shows the simulated morphological transition size from single to double layer (n = 18–19). **b-d**, Most stable morphology as a function of size and Pt–Al_2O_3 bond energy when the Pt–Pt bond energy is 0.5 eV (**b**), 0.98 eV (**c**), and 2 eV(**d**).

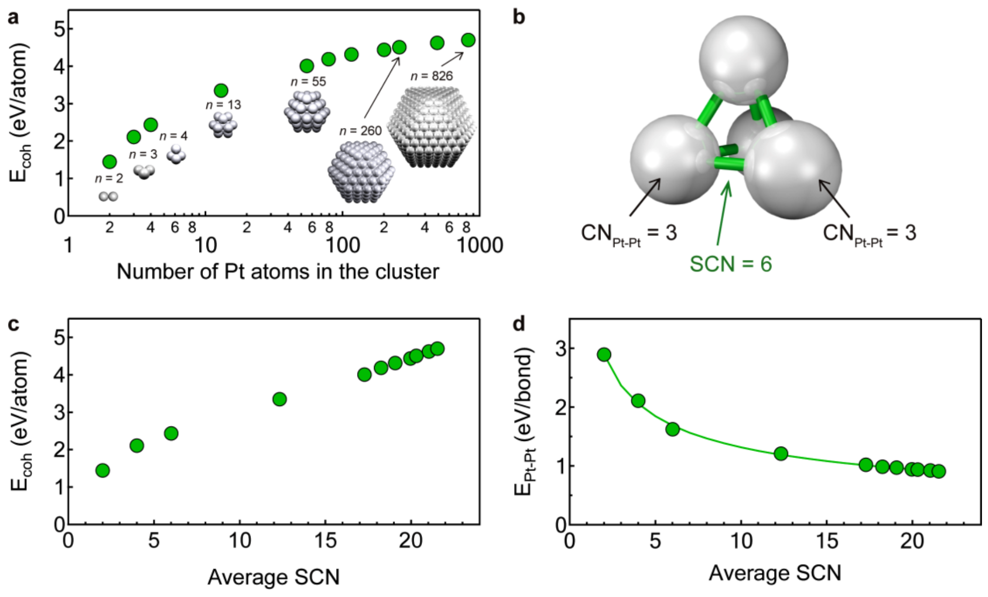

Supplementary Fig. 15 | Average Pt–Pt bond energy of freestanding Pt$_n$ clusters calculated using DFT. a, Cohesive energy of freestanding Pt$_n$ clusters as a function of size. Schematics in the figure show model Pt$_n$ structures used to calculate the cohesive energy. **b**, Schematic of Pt_4 tetrahedron, in which the coordination number ($CN_{Pt–Pt}$) of all Pt atoms is 3. A Pt–Pt bond was labelled by a sum of $CN_{Pt–Pt}$ (SCN) of Pt atoms at both ends of the Pt–Pt bond (SCN = 3 + 3 = 6). **c**, Cohesive energy of freestanding Pt$_n$ clusters as a function of average SCN, where average SCN was calculated by counting CN of all Pt atoms in the model Pt$_n$ cluster and labelling all Pt–Pt bonds by SCN, followed by dividing the sum of the SCN by the total number of Pt–Pt bonds. **d**, Average Pt–Pt bond energy as a function of average SCN, calculated by multiplying the cohesive energy by the number of Pt atoms in the cluster and dividing by the total number of Pt–Pt bonds. Solid line shows fitted line using Supplementary Equation 10. There was also an enhancement of bond energy between neighbouring Pt atoms (Pt–Pt); however, we have shown that it is not necessarily required to build the thermodynamically stable structural model for predicting optimum Pt cluster size for

CO oxidation.

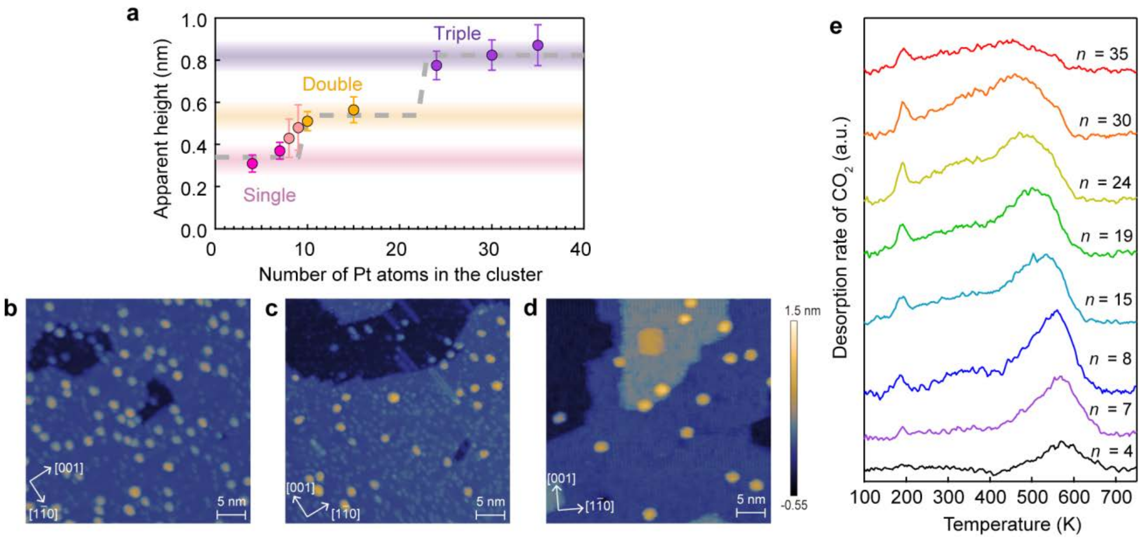

Supplementary Fig. 16 | Morphology and CO_2 TPR for Pt_n on TiO_2(110). a, Size dependency of average cluster height on TiO_2. Error bars and dashed line represent the standard deviation and simulated morphology from our modified BAM, respectively. **b-d**, STM images of Pt_n/TiO_2; n = 7 (**b**), n = 10 (**c**), and n = 24 (**d**). STM measurements were performed at 78 K. **e**, CO_2 TPR spectra (m/z = 47) over Pt_n/TiO_2. The sample surfaces were exposed to 1000 L of $^{18}O_2$ at 300 K followed by saturation adsorption of ^{13}CO at 88 K, and then, TPR measurements were performed.

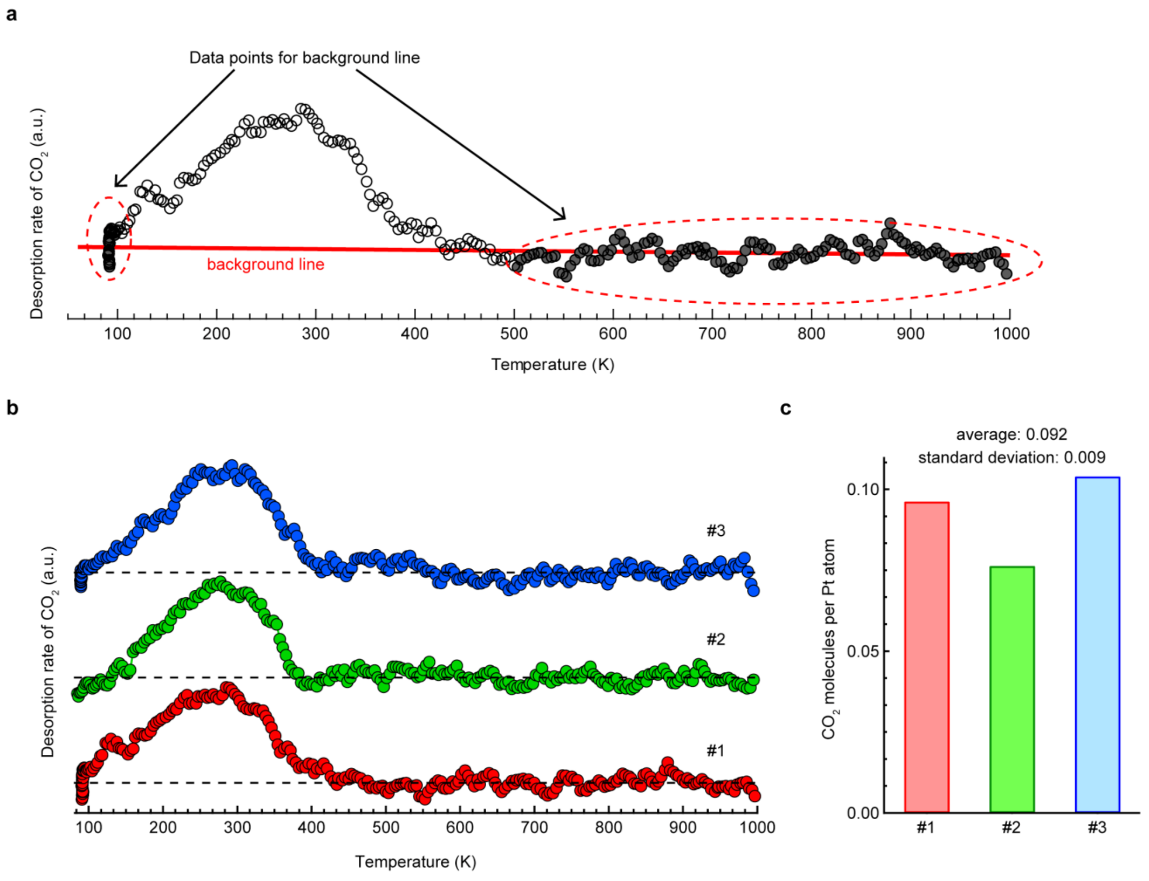

Supplementary Fig. 17 | Standard deviation of CO_2 TPR. **a**, Background subtraction from a measured CO_2 TPR spectrum over Pt_{30}/Al_2O_3. The red line shows the calculated background line, for which more than 10 data points from 100 K and below and from 500 K and above were linearly fitted by a least-square method. **b**, CO_2 TPR spectra over Pt_{30}/Al_2O_3. Three TPR experiments were performed in the same manner to quantitatively estimate experimental error. **c**, Number of CO_2 molecules (CO oxidation activity) calculated from the area intensity of the TPR spectra shown in (**b**). For the three CO_2 TPR spectra, the average and standard deviation of the number of CO_2 molecules were 0.092 and 0.009 CO_2 molecules/Pt atom, respectively.

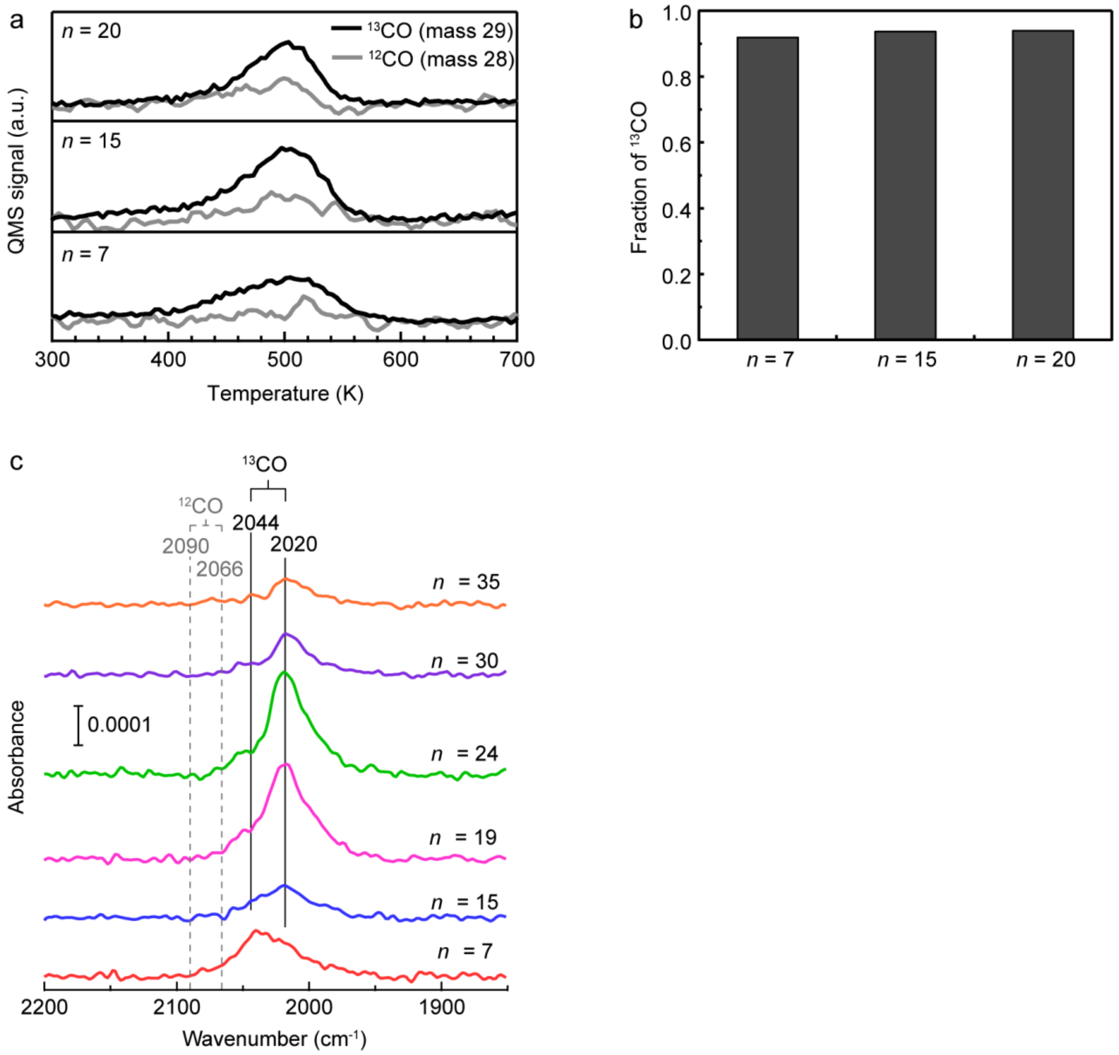

Supplementary Fig. 18 | Estimation of the amount of background ^{12}CO adsorbed on Pt_n/Al_2O_3. a, TPD spectra of background ^{12}CO and ^{13}CO and **b,** the fraction of ^{13}CO. **c,** IRAS spectra of ^{13}CO-saturated Pt_n/Al_2O_3. The peak position of ^{12}CO is indicated in grey, which was simply calculated by considering the isotopic shift.

Supplementary Table 1

Supplementary Table 1 | Reported vibrational frequency of ontop CO adsorbed on Pt catalysts

Structure of Pt catalyst	Oxidation state of Pt	Support material/substrate	Vibrational frequency (cm^{-1}) ^{12}CO [a]	^{13}CO [b]	Reference
Single atom	Pt(IV)	CeO_2	2089	2043	16
	Pt(IV)	FeO_x	2089	2043	17
	Pt(II)～Pt(IV)	SiO_2	2107	2060	18
	Pt(II)～Pt(IV)	Al_2O_3	2107	2060	18
	Pt(II)～Pt(IV)	TiO_2	2097	2050	18
	Pt(II)～Pt(IV)	TiO_2	2121	2074	18
	Pt(II)～Pt(IV)	ZrO_2	2088	2042	18
	Pt(II)	CeO_2	2095	2048	19
	Pt(II)	CeO_2	2098	2051	20
	Pt(II)	CeO_2	2090	2044	21
	Pt(II)	TiO_2	2112	2065	22
	Pt(II)	TiO_2	2110	2063	23
	Pt(II)	Al_2O_3	2110	2063	23
	Pt(0)～Pt(II)	θ-Al_2O_3	2058	2012	24
	Pt(0)～Pt(II)	Mesoporous Al_2O_3	2087	2041	25
	Pt(0)	Cu_2O/Cu(111)	2028	1983	15
Nanoparticle/cluster	Pt(IV)	CeO_2	2128	2081	26
	Pt(II)	Si	2078	2032	27
	Pt(II)	CeO_2	2102	2055	28
	Pt(II)	CeO_2	2120	2073	28
	Pt(II)	CeO_2	2122	2075	26
	Pt(0)～Pt(II)	TiO_2	2118	2071	22
	Pt(0)～Pt(II)	CeO_2	2090	2044	29
	Pt(0)～Pt(II)	CeO_2	2105	2058	29
	Pt(0)～Pt(II)	CeO_2	2086	2040	28
	Pt(0)～Pt(II)	CeO_2	2092	2046	28
	Pt(0)～Pt(II)	CeO_2	2096	2049	26
	Pt(0)	Cu_2O/Cu(111)	2065	2019	15
	Pt(0)	Cu_2O/Cu(111)	2045	2000	15
	Pt(0)	FeO_x	2030	1985	30
	Pt(0)	SiO_2,Al_2O_2,TiO_2,ZrO_2	2050	2004	18
	Pt(0)	SiO_2,Al_2O_2,TiO_2,ZrO_2	2080	2034	18
	Pt(0)	TiO_2	2040	1995	22

	Pt(0)	TiO_2	2090	2044	22
	Pt(0)	SiO_2	2094	2047	31
	Pt(0)	SiO_2	2076	2030	31
	Pt(0)	SiO_2	2046	2001	31
	Pt(0)	Al_2O_3	2080	2034	32
	Pt(0)	Al_2O_3	2098	2051	32
	Pt(0)	Al_2O_3	2060	2014	32
	Pt(0)	Al_2O_3	2075	2029	32
	Pt(0)	Al_2O_3	2070	2024	33
	Pt(0)	Al_2O_3	2090	2044	33
	Pt(0)	Si	2026	1981	27
	Pt(0)	Si	2061	2015	27
	Pt(0)	Si	2053	2007	27
	Pt(0)	Si	2048	2002	27
	Pt(0)	CeO_2	2053	2007	29
	Pt(0)	CeO_2	2066	2020	29
	Pt(0)	CeO_2	2080	2034	29
	Pt(0)	CeO_2	2097	2050	29
	Pt(0)	CeO_2	2066	2020	28
	Pt(0)	CeO_2	2085	2039	28
	Pt(0)	CeO_2	2086	2040	26
Bulk single crystal	Pt(0)～Pt(II)	p(2x2)-O/Pt(111)	2101	2054	34
	Pt(0)～Pt(II)	p(2x2)-O/Pt(111)	2109	2062	34
	Pt(0)	Pt(997)	2097	2050	35
	Pt(0)	Pt(997)	2083	2037	35
	Pt(0)	Pt(997)	2062	2016	35
	Pt(0)	Pt(997)	2071	2025	35
	Pt(0)	Pt(533)	2086	2040	36
	Pt(0)	Pt(533)	2097	2050	36
	Pt(0)	Pt(533)	2065	2019	36
	Pt(0)	Pt(533)	2078	2032	36
	Pt(0)	Pt(111)	2089	2043	34
	Pt(0)	Pt(111)	2105	2058	34
	Pt(0)	Pt(110)	2065	2019	37
	Pt(0)	Pt(110)	2094	2047	37

[a]Reported value; [b]Isotopically shifted value from reported ^{12}CO frequency.

Supplementary References

1. Heiz U., Sanchez A., Abbet S.&Schneider W.D. Catalytic Oxidation of Carbon Monoxide on Monodispersed Platinum Clusters: Each Atom Counts. *J. Am. Chem. Soc.* **121**, 3214-3217 (1999).

2. Häkkinen H., Abbet S., Sanchez A., Heiz U.&Landman U. Structural, Electronic, and Impurity-Doping Effects in Nanoscale Chemistry: Supported Gold Nanoclusters. *Angew. Chem.-Int. Edit.* **42**, 1297-1300 (2003).

3. Holmes Parker D., Jones M.E.&Koel B.E. Determination of the reaction order and activation energy for desorption kinetics using TPD spectra: Application to D2 desorption from Ag(111). *Surf. Sci.* **233**, 65-73 (1990).

4. Hopstaken M.J.P.&Niemantsverdriet J.W. Structure sensitivity in the CO oxidation on rhodium: Effect of adsorbate coverages on oxidation kinetics on Rh(100) and Rh(111). *J. Chem. Phys.* **113**, 5457-5465 (2000).

5. Miller J.B.*, et al.* Extraction of kinetic parameters in temperature programmed desorption: A comparison of methods. *J. Chem. Phys.* **87**, 6725-6732 (1987).

6. Nieskens D.L.S., van Bavel A.P.&Niemantsverdriet J.W. The analysis of temperature programmed desorption experiments of systems with lateral interactions; implications of the compensation effect. *Surf. Sci.* **546**, 159-169 (2003).

7. Yin C.*, et al.* Alumina-supported sub-nanometer Pt10 clusters: amorphization and role of the support material in a highly active CO oxidation catalyst. *J. Mater. Chem. A* **5**, 4923-4931 (2017).

8. Hoffmann F.M. Infrared reflection-absorption spectroscopy of adsorbed molecules. *Surf. Sci. Rep.* **3**, 107-192 (1983).

9. Vila F., Rehr J.J., Kas J., Nuzzo R.G.&Frenkel A.I. Dynamic structure in supported Pt nanoclusters: Real-time density functional theory and x-ray spectroscopy simulations. *Phys. Rev. B* **78**, 121404 (2008).

10. Kumar V.&Kawazoe Y. Evolution of atomic and electronic structure of Pt clusters: Planar, layered, pyramidal, cage, cubic, and octahedral growth. *Phys. Rev. B* **77**, 205418 (2008).

11. Rytkönen A., Valkealahti S.&Manninen M. Melting and evaporation of argon clusters. *J. Chem. Phys.* **106**, 1888-1892 (1997).

12. Jinnouchi R., Toyoda E., Hatanaka T.&Morimoto Y. First Principles Calculations on Site-Dependent Dissolution Potentials of Supported and Unsupported Pt Particles. *J. Phys. Chem. C* **114**, 17557-17568 (2010).

13. Kresse G., Schmid M., Napetschnig E., Shishkin M., Kohler L.&Varga P. Structure of the ultrathin aluminum oxide film on NiAl(110). *Science* **308**, 1440-1442 (2005).

14. Kaden W.E., Kunkel W.A., Kane M.D., Roberts F.S.&Anderson S.L. Size-Dependent Oxygen Activation Efficiency over Pdn/TiO2(110) for the CO Oxidation Reaction. *J. Am. Chem. Soc.* **132**, 13097-13099 (2010).

15. Therrien A.J.*, et al.* An atomic-scale view of single-site Pt catalysis for low-temperature CO oxidation. *Nat Catal.* **1**, 192-198 (2018).

16. Wang C.*, et al.* Water-Mediated Mars–Van Krevelen Mechanism for CO Oxidation on Ceria-Supported Single-Atom Pt1 Catalyst. *ACS Catal.* **7**, 887-891 (2017).

17. Lang R.*, et al.* Non defect-stabilized thermally stable single-atom catalyst. *Nat. Commun.* **10**, 234 (2019).

18. Ding K.*, et al.* Identification of active sites in CO oxidation and water-gas shift over supported Pt catalysts. *Science* **350**, 189-192 (2015).

19. Jones J.*, et al.* Thermally stable single-atom platinum-on-ceria catalysts via atom trapping. *Science* **353**, 150-154 (2016).

20. Nie L.*, et al.* Activation of surface lattice oxygen in single-atom Pt/CeO₂ for low-temperature CO oxidation. *Science* **358**, 1419-1423 (2017).

21. Chen J., *et al.* Surface Engineering Protocol To Obtain an Atomically Dispersed Pt/CeO2 Catalyst with High Activity and Stability for CO Oxidation. *ACS Sustain. Chem. Eng.* **6**, 14054-14062 (2018).

22. DeRita L., *et al.* Catalyst Architecture for Stable Single Atom Dispersion Enables Site-Specific Spectroscopic and Reactivity Measurements of CO Adsorbed to Pt Atoms, Oxidized Pt Clusters, and Metallic Pt Clusters on TiO2. *J. Am. Chem. Soc.* **139**, 14150-14165 (2017).

23. Liu S., *et al.* Stabilizing Single-Atom and Small-Domain Platinum via Combining Organometallic Chemisorption and Atomic Layer Deposition. *Organometallics* **36**, 818-828 (2017).

24. Moses-DeBusk M., *et al.* CO Oxidation on Supported Single Pt Atoms: Experimental and ab Initio Density Functional Studies of CO Interaction with Pt Atom on θ-Al2O3(010) Surface. *J. Am. Chem. Soc.* **135**, 12634-12645 (2013).

25. Lou Y.&Liu J. CO Oxidation on Metal Oxide Supported Single Pt atoms: The Role of the Support. *Ind. Eng. Chem. Res.* **56**, 6916-6925 (2017).

26. Daniel D.W. Infrared studies of carbon monoxide and carbon dioxide adsorption on platinum/ceria: the characterization of active sites. *J. Phys. Chem.* **92**, 3891-3899 (1988).

27. Wang H., *et al.* Influence of Size-Induced Oxidation State of Platinum Nanoparticles on Selectivity and Activity in Catalytic Methanol Oxidation in the Gas Phase. *Nano Lett.* **13**, 2976-2979 (2013).

28. Ke J., *et al.* Strong Local Coordination Structure Effects on Subnanometer PtOx Clusters over CeO2 Nanowires Probed by Low-Temperature CO Oxidation. *ACS Catal.* **5**, 5164-5173 (2015).

29. Happel M., *et al.* Adsorption sites, metal-support interactions, and oxygen spillover identified by vibrational spectroscopy of adsorbed CO: A model study on Pt/ceria catalysts. *J. Catal.* **289**, 118-126 (2012).

30. Qiao B., *et al.* Single-atom catalysis of CO oxidation using Pt1/FeOx. *Nat Chem.* **3**, 634 (2011).

31. Lundwall M.J., McClure S.M.&Goodman D.W. Probing Terrace and Step Sites on Pt Nanoparticles Using CO and Ethylene. *J. Phys. Chem. C* **114**, 7904-7912 (2010).

32. Kale M.J.&Christopher P. Utilizing Quantitative in Situ FTIR Spectroscopy To Identify Well-Coordinated Pt Atoms as the Active Site for CO Oxidation on Al2O3-Supported Pt Catalysts. *ACS Catal.* **6**, 5599-5609 (2016).

33. Allian A.D., *et al.* Chemisorption of CO and Mechanism of CO Oxidation on Supported Platinum Nanoclusters. *J. Am. Chem. Soc.* **133**, 4498-4517 (2011).

34. Yoshinobu J.&Kawai M. Thermal excitation of oxygen species as a trigger for the CO oxidation on Pt(111). *J. Chem. Phys.* **103**, 3220-3229 (1995).

35. Yoshinobu J., Tsukahara N., Yasui F., Mukai K.&Yamashita Y. Lateral displacement by transient mobility in chemisorption of CO on Pt(997). *Phys. Rev. Lett.* **90**, 248301 (2003).

36. Hayden B.E., Kretzschmar K., Bradshaw A.M.&Greenler R.G. An infrared study of the adsorption of CO on a stepped platinum surface. *Surf. Sci.* **149**, 394-406 (1985).

37. Klünker C., Balden M., Lehwald S.&Daum W. CO stretching vibrations on Pt(111) and Pt(110) studied by sumfrequency generation. *Surf. Sci.* **360**, 104-111 (1996).
